# Distinct Functional Connectivity Patterns of Brain Networks Involved in Motor Planning Underlie Verbal and Spatial Working Memory

**DOI:** 10.1002/brb3.70376

**Published:** 2025-02-28

**Authors:** Emilie Marti, Sélim Yahia Coll, Naz Doganci, Radek Ptak

**Affiliations:** ^1^ Faculty of Medicine University of Geneva Geneva Switzerland; ^2^ University Service of Neuropsychology and Neurorehabilitation, Department of Clinical Neuroscience University Hospitals of Lausanne Lausanne Switzerland; ^3^ Division of Neurorehabilitation, Department of Clinical Neurosciences Geneva University Hospitals Geneva Switzerland

**Keywords:** functional connectivity, motor planning, N‐back task, resting‐state fMRI, working memory

## Abstract

**Purpose:**

Frontoparietal networks (FPN) are well‐recognized for their role in high‐level cognition, including mental imagery, executive control, and working memory (WM). A prevailing hypothesis advances that these functions evolved from fundamental motor abilities, such as action planning and motor control. However, whether sensorimotor regions of these FPN contribute to the executive components of WM, and whether this contribution is dependent on task modality, remains underexplored.

**Method:**

This study applied analyses of resting‐state functional connectivity (rs‐FC) to investigate the contribution of FPN regions to WM that have an established role in motor planning. In a sample of 60 healthy individuals, we explored whether performance in verbal and spatial N‐back WM tasks is associated with rs‐FC of frontoparietal brain regions that exhibit increased activation during motor planning.

**Finding:**

Comparing verbal and spatial N‐back tasks revealed that verbal WM was associated with stronger connectivity between the left medial superior frontal gyrus and left inferior parietal lobule (IPL), as well as the right IPL and the left superior parietal lobule. In contrast, spatial WM was linked to stronger connectivity between the right middle frontal and inferior temporal gyrus, as well as the left occipital pole and postcentral gyrus.

**Conclusion:**

These findings reveal distinct FC patterns underlying verbal and spatial WM and highlight the contribution of brain regions that are important for motor planning to modality‐specific WM processes, such as information updating.

## Graphical abstract

This study investigates resting‐state functional connectivity associated with working memory performance in verbal and spatial N‐back tasks. We identified distinct functional connectivity patterns within frontoparietal regions involved in motor planning that underlie verbal and spatial working memory performance, highlighting the critical role of motor‐related regions in executive processes associated with mental updating of information.

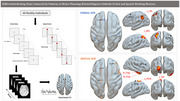

## 1 Introduction

A fundamental debate in cognitive neuroscience relates to the question whether cognitive functions are localizable within discrete cortical areas or distributed across brain networks. While some researchers have historically supported a localizationist perspective, arguing that specific cognitive functions are confined to distinct brain regions (Coltheart et al. 2001; Fodor 1983; Shallice and Cooper 2012), evidence from functional neuroimaging increasingly challenges this view. A growing number of studies show that large‐scale brain networks engage in a wide range of cognitive processes, suggesting that cognition arises from complex interactions among these networks (Anderson et al. 2013).

Theories of neural reuse provide a compelling framework for understanding how large‐scale networks facilitate various cognitive faculties. This perspective conceptualizes the brain as a flexible, dynamic system composed of adaptable neural networks that harbor distributed and embodied cognitive representations (Anderson 2010). Neural reuse specifically refers to the brain's ability to repurpose its structures and resources in response to changing environmental demands, thereby maintaining their availability for original functions while supporting new cognitive activities (Anderson 2007). Rather than simply optimizing existing cognitive patterns, the brain can reorganize primitive circuit features to develop new and more complex functions within established frameworks (Anderson 2014; Dehaene and Cohen 2007). Consequently, complex cognition can be seen as emerging from the expression of elementary core functions in novel contexts (Ptak et al. 2021; Spelke and Kinzler 2007).

This reorganization is evident in an early development of sensory and motor networks, which suggests that primitive cognitive networks may have evolved from these pre‐existing sensorimotor sources (Buckner and Krienen 2013; Koziol and Lutz 2013). Indeed, developmental studies indicate a strong link between motor control and the emergence of executive functions; for instance, research shows that the kinematics of reaching movements can predict working memory (WM) and response inhibition in 18‐month‐old toddlers (Gottwald et al. 2016). Additionally, evidence of significant connectivity between motor and cognitive structures suggests that these anatomical connections transmit shared signals (Chein and Schneider 2005; Duncan and Owen 2000). This interdependence implies that cognitive development is closely tied to the re‐enactment of sensorimotor experiences (Barsalou 2008; Thelen 2000), suggesting that high‐level functions emerge from the recombination of motor and object representations sharing similar neural resources (McIntosh 2000).

In this context, resting‐state functional magnetic resonance imaging (rs‐fMRI) has emerged as a powerful tool for identifying large‐scale network interactions in the human brain by analyzing low‐frequency BOLD signal fluctuations during rest, a method known as functional connectivity (FC) (Biswal et al. 1995; Fair et al. 2007; Fox and Raichle 2007). FC reflects the functional collaboration between distant brain regions, with its relevance to behavior corroborated by findings linking FC to task performance in healthy participants and individuals with brain injuries.

This perspective provides valuable insight into the roles and activations of high‐scale networks, particularly frontoparietal networks (FPN) involving the dorsal prefrontal cortex (PFC) and posterior parietal cortex (PPC) (Ptak 2012). FPN are particularly notable for their adaptability across a range of motor and cognitive tasks, including WM (Rottschy et al. 2012), mental imagery (Zacks 2008), and executive control (Cole and Schneider 2007). Evidence from patient studies reveals that lesions in FPN can disrupt motor and cognitive functions, suggesting that these networks have evolved into general domain networks capable of integrating diverse processes, such as motor actions and their mental manipulation and maintenance (Ptak et al. 2017). A hypothesis put forward to explain this overlap is that high‐level functions engaging FPN share a core component rooted in elementary motor processes, such as motor planning (Ptak et al. 2021).

Among the cognitive functions that heavily rely on the PFC and PPC is the maintenance and manipulation of mental information, a defining purpose of WM (Owen et al. 2005; Pessoa et al. 2002). WM is often conceptualized as a central executive system responsible for allocating attentional resources, managing information manipulation, and overseeing two storage systems: a phonological loop for verbal information and a visuo‐spatial sketchpad for visual information (Baddeley and Hitch 1974). Given the limitations of passive storage and attentional resources, additional strategies are necessary for retaining information, such as updating of representations as new material is presented (e.g., in N‐back tasks), and actively rehearsing verbal (Marvel and Desmond 2012) or nonverbal content (Hétu et al. 2013). Tasks designed to assess the executive demands of WM, particularly the updating component required in N‐back paradigms, demonstrate dorsal frontoparietal activation patterns across both hemispheres during verbal and nonverbal rehearsal (Cowan et al. 2011; Nystrom et al. 2000; Rottschy et al. 2012). Lesion studies further support a causal involvement of frontoparietal areas in WM, showing that damage to these regions significantly affects WM performance in brain‐injured patients (Baldo and Dronkers 2006; Barbey et al. 2013; Marti et al. 2024; Martin et al. 2021; Ptak and Schnider 2004; Tsuchida and Fellows 2009; van Asselen et al. 2006). These findings underscore the critical role of frontoparietal areas in WM and suggest that the functional connectivity within this network may contribute to performance on WM tasks.

Additionally, neuroimaging data suggest that WM is also supported by regions traditionally associated with motor functions, such as the premotor cortex (PMC), presupplementary motor area (preSMA), and supplementary motor area (SMA) (Champod and Petrides 2007; Chein and Fiez 2001; Wager and Smith 2003). These areas show increased activation prior to motor execution, even in the absence of overt movements, indicating a supportive role in motor planning and preparation (Hülsmann et al. 2003). Furthermore, tasks involving the manipulation of verbal and nonverbal mental contents elicit heightened activity across the PMC (Marvel and Desmond 2012), suggesting that motor representations significantly contribute to the maintenance of information in WM (Leisman et al. 2016). Finally, inhibitory transcranial magnetic stimulation of the PMC interferes with verbal and non‐verbal mental rehearsal, suggesting a causal involvement of the PMC in WM (Liao et al. 2014).

Despite these findings, the contribution of brain regions involved in motor planning to WM remains poorly understood, particularly regarding executive WM components such as updating. To address this gap, the present study investigated the associations between frontoparietal connectivity and WM, using insights from resting‐state functional connectivity (rs‐FC). In a sample of 60 healthy individuals, we examined whether behavioral performance in verbal and spatial N‐back tasks was associated with rs‐FC of brain regions that exhibit increased activation during motor planning. The aims of our study were to determine whether rs‐FC patterns in regions associated with motor preparation correlate with WM and to explore potential modality‐specific connectivity patterns underlying the manipulation and updating of verbal and spatial information.

## 2 Materials and Methods

### 2.1 Participants

Prior to participation, all individuals provided informed consent, and the study received ethical approval from the Canton of Geneva's ethics committee, aligning with the principles of the Declaration of Helsinki.

The study initially involved a sample of 85 healthy right‐handed participants. The inclusion criteria entailed right‐handedness, the absence of current or previous neurological or psychiatric disorders, and compatibility with MRI scanning procedures. Six participants were excluded from the analyses due to testing or MRI scanning issues like excessive head movements or missing behavioral or functional data. Additionally, nineteen participants out of the initial sample refused to participate to the fMRI part of the experimental protocol. Consequently, 60 right‐handed healthy controls (mean age = 49 ± 19.4 years, 27 female) successfully completed both the behavioral and functional experiments and were included in the analysis. The study involved two distinct sessions: one behavioral, focusing on assessments and testing outside the MRI scanner, and an MRI session for performing the rs‐fMRI. The behavioral session evaluated participants' WM performance in both verbal and spatial N‐back tasks.

### 2.2 Behavioral Tasks

**FIGURE 1:**
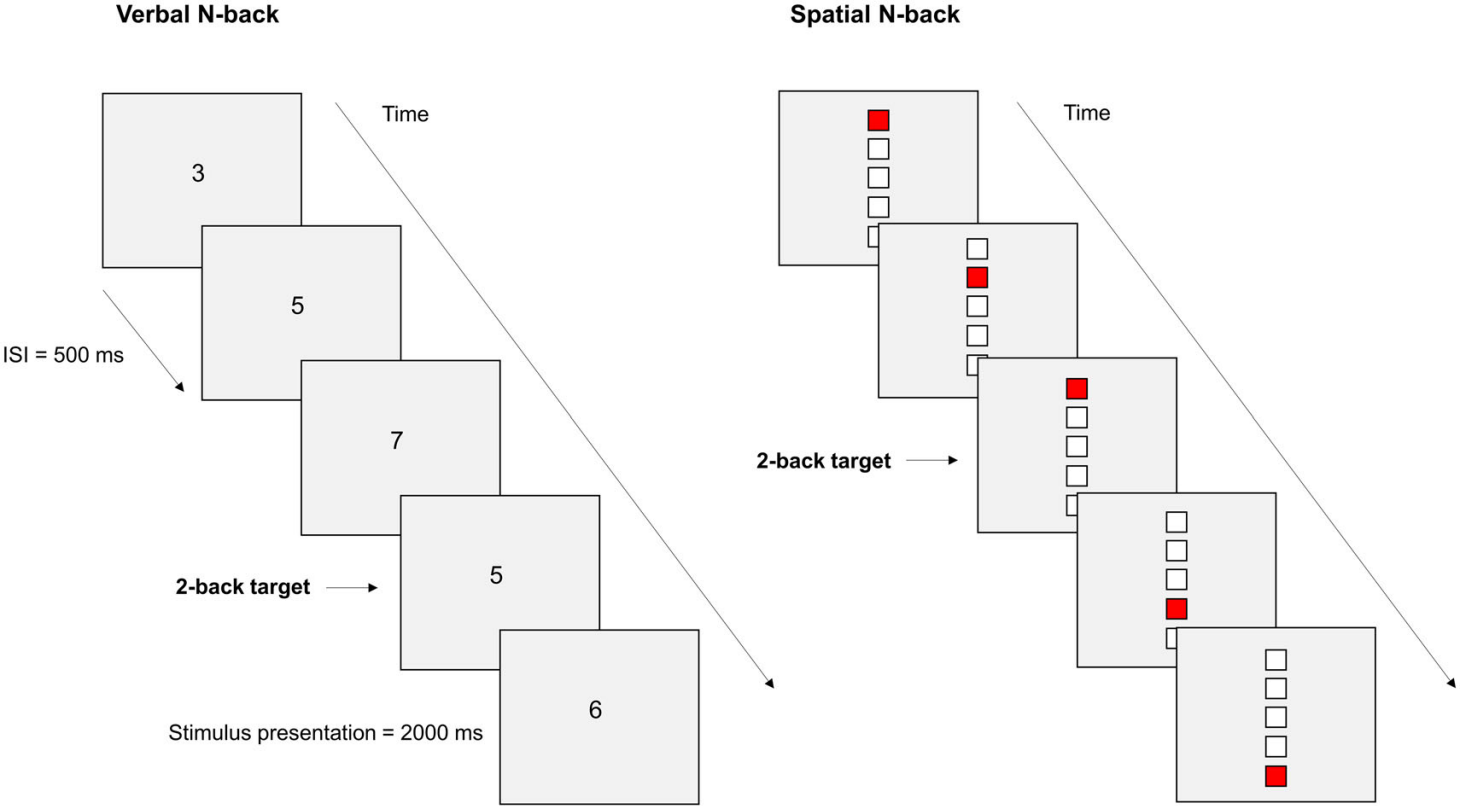
Illustration of the N‐back tasks. Left: Verbal N‐back task, where a series of 5 digits (e.g., 3, 4, 5, 6, and 7) is presented in pseudo‐random order. Participants are instructed to press a button when the current digit matches the one presented two positions earlier (2‐back target). Right: Spatial N‐back task, where 5 blank squares are vertically arranged. Participants respond when a red square reappears in the same position as two trials before (2‐back target). Each stimulus is displayed for 2000 ms, followed by a 500 ms interstimulus interval (ISI).

WM performance was assessed using two continuous recognition tasks based on the N‐back paradigm (Kirchner 1958), requiring participants to determine whether the current item matches the item presented N steps earlier in a sequence of verbal or spatial stimuli. The experimental task, developed using E‐Prime 3.0 software (Psychology Software Tools, Pittsburgh, PA), was presented on a 13.3‐inch HP touchscreen laptop positioned 50 cm from the subjects. Participants completed an adaptation of N‐back for verbal and spatial information separately (Figure 1). In the verbal 2‐back, a continuous series consisting of the digits 3, 4, 5, 6, and 7 appeared in a pseudo‐random order, and subjects were instructed to press the button on the response box (Cedrus, San Pedro, CA) when the same digit occurred two positions before. The spatial 2‐back task involved a vertical arrangement of five blank squares. During each trial, one square turned red. Participants had to respond if the red square appeared in the same position as it did two trials before.

Participants were required to respond within a 2‐s window, corresponding to the duration of stimulus presentation. They completed 124 trials (62 trials across two blocks) for each 2‐back condition (verbal and spatial). Responses were classified into hits (correct responses to targets), false alarms (responses to distractors), misses (either nonresponses or responses exceeding the time limit for targets), and correct rejections (no response to distractors). Recording misses allowed for the capture of both nonresponses and timing‐related errors.

To evaluate N‐back task accuracy, the discriminability index was used (d′; Haatveit et al. 2010), derived from Signal Detection Theory. This index integrates both correct detections (hits) and incorrect responses (false alarms) to assess participants' ability to discriminate between targets and distractors. The calculation, d′ = Zhits−Zfalse alarms, provides a higher value for greater discrimination accuracy, reflecting better WM performance.

### 2.3 Resting‐State fMRI

#### 2.3.1 Neuroimaging Acquisition

Structural and functional magnetic resonance imaging, including an rs‐fMRI scan, were performed in a single session on a Siemens 3T Prisma fit scanner with a 64‐channel array coil (Siemens Medical Solutions, Erlangen, Germany) located at the University Hospitals of Geneva (HUG, Geneva, Switzerland).

High‐resolution T1‐weighted MPRAGE sequences were utilized to acquire whole‐brain structural images (TR: 2300 ms; TE: 1.96 ms; number of images = 176; voxel size = 1.0 mm isometric; flip angle = 9°). Functional images were generated using a fast Echoplanar Imaging sequence (TR: 720 ms; TE: 30 ms; number of images = 440; voxel size = 2.5 mm isometric; flip angle = 50°). During acquisition, the subject's head was stabilized with cushions to prevent movement. Participants laid still in the dark, with open eyes staring at an MRI‐compatible screen showing a centered white fixation cross on a black background.

#### 2.3.2 rs‐fMRI Data Processing

Anatomical and functional data were processed using a flexible preprocessing pipeline in the CONN toolbox (Whitfield‐Gabrieli and Nieto‐Castanon 2012). Functional data were realigned: all scans were coregistered to a reference image and resampled to correct for motion and magnetic susceptibility interactions. Temporal misalignment between different slices was corrected using sinc temporal interpolation to resample each slice BOLD time series to a common mid‐acquisition time. Outlier scans were identified using ART (Power et al. 2014), and a reference BOLD image was computed for each subject by averaging all scans excluding outliers. Normalization of anatomical and functional data into standard MNI space was achieved through SPM (SPM12; http://www.fil.ion.ucl.ac.uk/spm) unified segmentation and normalization algorithm, followed by smoothing with an 8 mm FWHM Gaussian kernel. Additionally, denoising was performed using a standard pipeline, including regression of potential confounding effects such as white matter and CSF timeseries, motion parameters, outlier scans, session effects, and linear trends within each functional run. Bandpass frequency filtering was applied to the BOLD timeseries between 0.008 and 0.09 Hz. CompCor noise components (Behzadi et al. 2007) within white matter and CSF were estimated, contributing to the effective degrees of freedom estimation of the BOLD signal after denoising. FC strength was represented by Fisher‐transformed bivariate correlation coefficients using a weighted general linear model (GLM; Nieto‐Castanon 2020). Group‐level analyses utilized a GLM with voxel‐level hypotheses evaluated using multivariate parametric statistics, followed by cluster‐level inferences based on Gaussian Random Field theory. Results were thresholded using a combination of a cluster‐forming *p* < 0.001 voxel‐level threshold, and a familywise corrected *p*‐FDR < 0.05 cluster‐size threshold.

#### 2.3.3 Seed‐Based FC Analyses

To investigate our hypothesis that motor planning‐related regions also contribute to WM, we targeted specific cortical seed regions within FPN: the dorsolateral prefrontal cortex (dlPFC), dorsomedial prefrontal cortex (dmPFC), and PPC (Owen et al. 2005; Rottschy et al. 2012; Wager and Smith 2003). We defined 10 regions of interest (ROIs) derived from a previous study that examined motor planning processes with a task contrasting internally and externally triggered finger movements (Doganci et al. 2023). In the latter, participants received a visual cue indicating which finger they had to move (e.g., the middle finger) out of four possibilities (index, middle, ring, and little finger). In the internally triggered condition, they decided spontaneously which finger to move. By contrasting internally with externally triggered finger movements, they identified brain regions implicated in motor planning related to a specific effector. This comparison highlighted two distinct networks: a frontoparietal and a temporo‐occipital network. For the present study, we focused specifically on the frontoparietal regions. Accordingly, the selected ROIs were concentrated on the lateral and medial surfaces of the frontoparietal cortex, primarily within the superior and middle frontal gyri, as well as the inferior and superior parietal lobes. More precisely, the regions included the bilateral middle frontal gyrus (MFG), the medial superior frontal gyrus (mSFG) corresponding to the SMA, bilateral superior frontal gyrus (SFG), bilateral inferior parietal lobule (IPL), and bilateral superior parietal lobule (SPL).

We first analyzed FC patterns within each modality separately to investigate the relationship between connectivity and WM performance. To further align with the study's aim of identifying modality‐specific connectivity, we then conducted a unified analysis that directly contrasted verbal and spatial WM. These analyses allowed us to identify distinct correlations between FC and d’ by comparing directly the two modalities. Specifically, the *Verbal* > *Spatial* contrast highlights FC patterns that are positively associated with verbal WM (higher FC = higher d′) and negatively associated with spatial WM (higher FC = lower d′), while the *Spatial* > *Verbal* contrast reflects the opposite pattern. Both contrasts therefore capture connectivity differences that favor the involvement in processing demands of one modality over the other. To identify FC correlates of behavioral scores, we calculated the average connectivity values for each significant cluster and computed the d′ deltas between the two modalities, as represented by the contrast. We then examined the relationships between these connectivity measures and behavioral scores using Pearson correlations.

While our focus was on cortical ROIs, we systematically computed seed‐based FC across the entire brain, including subcortical regions. It is important to note that any involvement of these subcortical regions would consequently be detected within the framework of our analytical approach.

## 3 Results

### 3.1 Behavioral Results

In the 2‐back tasks, participants achieved an average accuracy of 93.6% for verbal WM (20 hits, 96 correct rejections, 4 misses, and 4 false alarms) and 92.7% for spatial WM (20 hits, 95 correct rejections, 5 misses, and 4 false alarms). Descriptive statistics indicated that participants, on average, exhibited a higher d′ in verbal WM (*M* = 3.10, SD = 1.06) compared to spatial WM (*M* = 2.98, SD = 1.06).

Behavioral data were analyzed using Rstudio (Rstudio Team 2023). A linear mixed model was employed with d′ as the dependent variable. The model incorporated the Modality of the task (Verbal, Spatial) as a fixed factor, while subjects and age were treated as random factors. The analysis revealed no significant difference in d' scores between Verbal and Spatial WM [*F*(1, 59) = 0.914, *p* = 0.343)].

### 3.2 FC Predictors of WM

Seed‐based regression analyses were conducted to identify the brain regions where the FC with pre‐defined ROIs shows significant correlation with verbal and spatial WM performance, as measured by d′. Given that our sample had a large age range, and we were not interested in age‐related differences, age was included as a covariate in the regression model.

When analyzed separately, there was a significant negative correlation between verbal d′ and FC between the left mSFG (corresponding to the SMA) and the triangular part of the left inferior frontal gyrus (IFG) (cluster size: 209 voxels; MNI coordinates: −50, +38, −02; FDR‐corrected *p* = 0.004). Analyses of spatial WM yielded a significant negative correlation between spatial d′ and FC strength between the right IPL and the right cerebellum (cluster size: 168 voxels; MNI coordinates: +10, −92, −30; FDR‐corrected *p* = 0.045). Note that a negative correlation indicates that higher FC between these regions is associated with lower WM performance in each modality.

**FIGURE 2:**
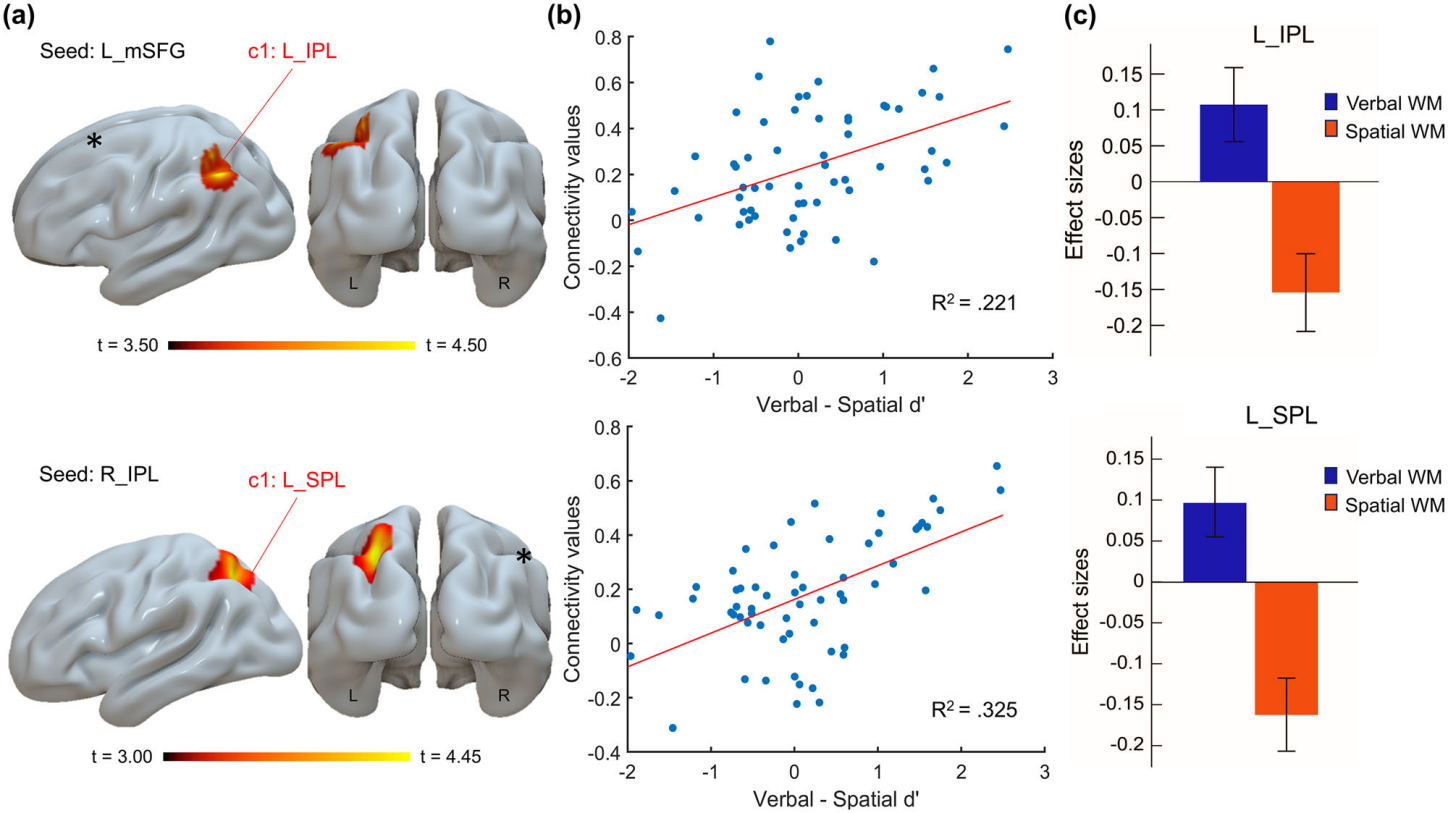
Results of global seed‐based connectivity analyses for the contrast *Verbal* > *Spatial*. (a) Clusters where functional connectivity with the left middle superior frontal gyrus (mSFG; top) and right inferior parietal lobule (IPL; bottom) seeds showed a stronger positive correlation with verbal d′ compared to spatial WM. (b) Scatterplots depicting the correlation between connectivity values (averaged across all voxels within the identified clusters) and the difference in performance (verbal d′–spatial d′), representing the contrast of interest. (c) Effect sizes for the seed‐based functional connectivity analysis between the verbal and spatial modalities (seeds are indicated by asterisks). IPL indicates inferior parietal lobe; mSFG, medial superior frontal gyrus; SPL, superior parietal lobe.

For the contrast *Verbal* > *Spatial*, two brain regions exhibited significantly greater positive behavior‐FC associations for verbal compared than spatial WM. The first was between the left mSFG (corresponding to the SMA) and the left IPL (cluster size: 303 voxels; MNI coordinates: −46, −58, +56; FDR‐corrected *p* > 0.001; Figure 2a). The second was between the right IPL and the left superior parietal lobule (SPL; cluster size: 274 voxels; MNI coordinates: −26, −58, +42; FDR‐corrected *p* = 0.002; Figure 2b). To further explore these findings, Pearson correlations were computed between FC strength and the difference between verbal and spatial d′. Moderate positive correlations were identified for left mSFG–left IPL connectivity (*r* = 0.467, *p* < 0.001) and right IPL–left SPL connectivity (*r* = 0.567, *p* < 0.001). These results indicate that higher FC in these regions is associated with higher verbal d′ (i.e., predicting better performance) relative to spatial WM.

**FIGURE 3:**
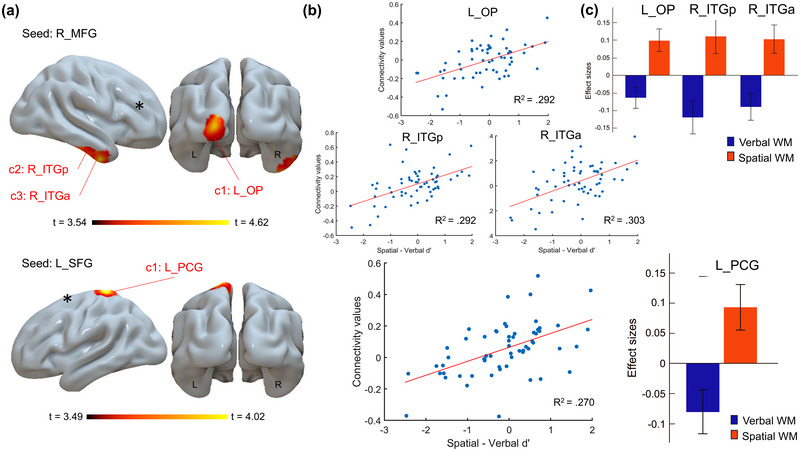
Results of global seed‐based connectivity analyses for the contrast *Spatial* > *Verbal*. (a) Clusters where functional connectivity with the right middle frontal gyrus (MFG; top) and left superior frontal gyrus (SFG; bottom) seeds showed a stronger positive correlation with spatial d′ compared to verbal WM (c1 = cluster 1, c2 = cluster 2, c3 = cluster 3). (b) Scatterplots depicting the correlation between connectivity values (averaged across all voxels within the identified clusters) and the difference in performance (spatial d′–verbal d′), representing the contrast of interest. (c) Effect sizes for the seed‐based FC analysis between the verbal and spatial modalities (seeds are indicated by asterisks). ITG indicates inferior temporal gyrus; MFG, middle frontal gyrus; OP, occipital pole; PCG, postcentral gyrus; SFG, superior frontal gyrus.

For the contrast *Spatial* > *Verbal*, significant positive FC was observed between the right MFG and three distinct clusters (Figure 3a). These clusters were localized in the left occipital pole (OP; cluster size: 171 voxels; MNI coordinates: −24, −100, +08; FDR‐corrected *p* = 0.026), the right posterior inferior temporal gyrus (ITG; cluster size: 115 voxels; MNI coordinates: +58, −20, −30; FDR‐corrected *p* = 0.042) and the right anterior ITG (cluster size: 108 voxels; MNI coordinates: +48, −04, −32; FDR‐corrected *p* = 0.042), respectively. Additionally, FC between the left SFG and the left postcentral gyrus (PCG; cluster size: 127 voxels; MNI coordinates: −18, −36, +68; FDR‐corrected *p* = 0.038) exhibited a significant positive correlation with higher spatial d′ (Figure 3b).

Finally, moderate correlations were also observed between FC and spatial‐verbal differences in d′ for MFG‐OP (*r* = 0.540, *p* < 0.001), MFG‐posterior ITG (*r* = 0.535, *p* < 0.001), MFG‐anterior ITG (*r* = 0.546, *p* < 0.001), and SFG‐PCG connectivity (*r* = 0.522, *p* < 0.001). These findings suggest that higher FC in these regions is associated with higher spatial d′, while simultaneously corresponding to lower verbal d′.

## 4 Discussion

**FIGURE 4:**
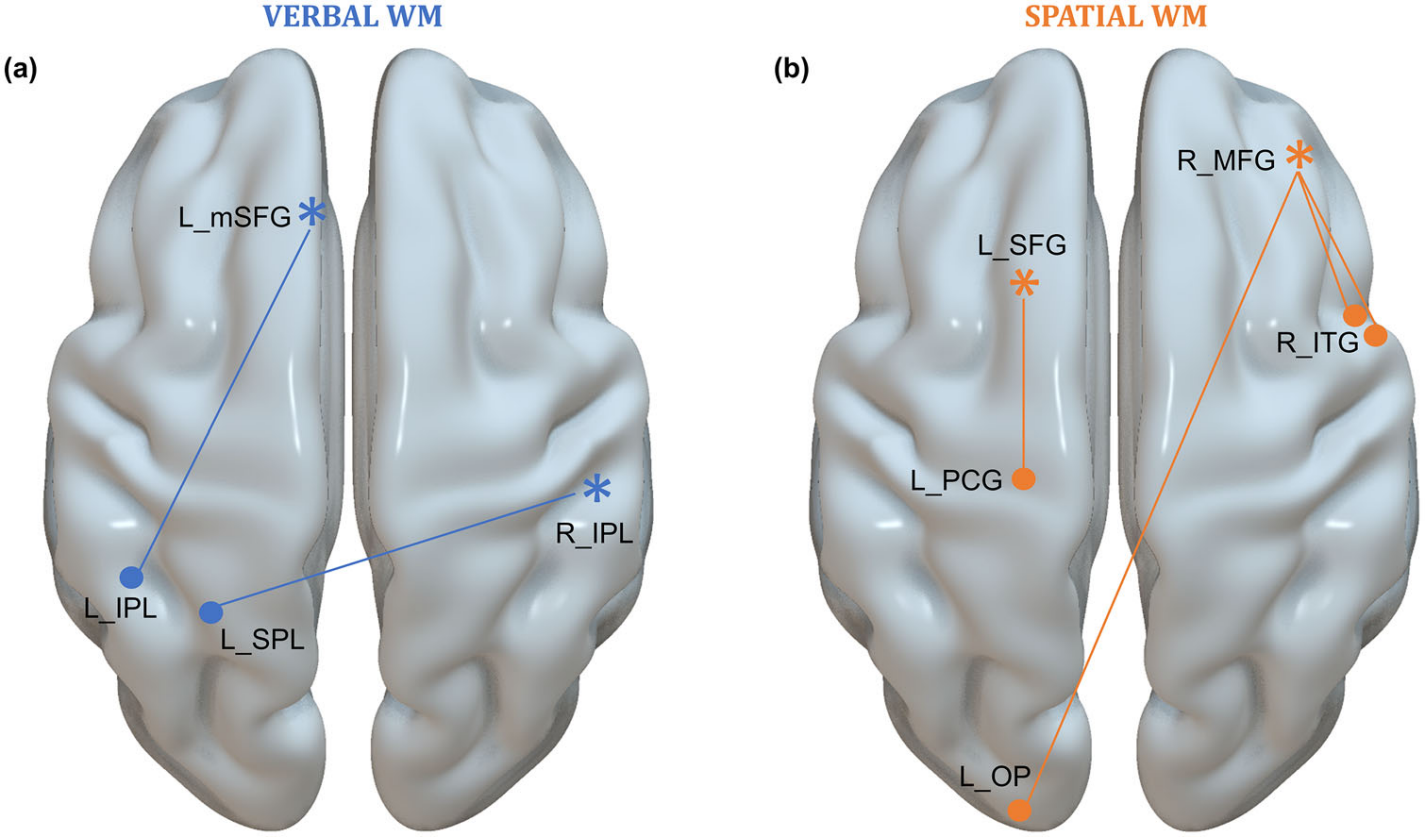
Distinct resting‐state networks whose functional connectivity with seed regions correlated with WM performance. (a) Resting‐state network exhibiting greater positive correlations during rest with verbal WM. (b) Resting‐state network exhibiting greater positive correlation during rest with spatial WM (seeds are indicated by asterisks). IPL indicates inferior parietal lobe; ITG, inferior temporal gyrus; MFG, middle frontal gyrus; mSFG, medial superior frontal gyrus; OP, occipital pole; PCG, postcentral gyrus; SFG, superior frontal gyrus; SPL, superior parietal lobe.

Our study aimed to investigate the associations between rs‐FC in brain regions that are important for motor planning and performance on verbal and spatial N‐back tasks, with a particular focus on distinct connectivity patterns between the two modalities. The results showed that WM scores significantly correlated, either positively or negatively, with rs‐FC among regions within the dlPFC, PMC, and PPC, exhibiting distinct patterns for verbal and spatial WM (Figure 4). More precisely, comparisons of verbal and spatial WM showed that verbal WM was positively associated with connectivity between the left mSFG and left IPL, as well as the right IPL and left SPL (Figure 2). In contrast, spatial WM correlated positively with connectivity involving the right MFG, right ITG, left OP, and left PCG (Figure 3). These observations align with previous rs‐FC studies, which showed that the functional organization at rest is strongly associated with behavioral performance and shares common structural foundations (Hermundstad et al. 2013; Mennes et al. 2010, 2011). Specifically, several studies have identified frontoparietal regions as predictors of WM capacity and performance (Hampson et al. 2006; Osaka et al. 2021), particularly in complex tasks such as the N‐back (Liu et al. 2017; Zou et al. 2013). At rest, spontaneous brain activity organizes into networks that closely mirror those activated during task performance, suggesting that rs‐activity may facilitate the engagement of brain networks required for task execution and responses to external stimuli (Smith et al. 2009). Consistent with previous descriptions of key regions involved in the N‐back task (Owen et al. 2005), our results suggest that brain networks critical for the maintenance, manipulation, and updating of information in WM functionally overlap with brain regions involved in motor planning.

### 4.1 Distinct rs‐Networks Supporting Verbal and Spatial WM

The involvement of brain regions whose FC positively correlates with verbal d′ aligns closely with established models of verbal information processing in WM. Specifically, the involvement of the left SMA has been associated with articulatory rehearsal, while the left IPL has been linked to phonological storage (Chein and Fiez 2001; Henson et al. 2000; Paulesu et al. 1993; Smith et al. 1998). A recent study demonstrated that strong connectivity between the medial PFC and PPC, both during task performance and at rest, is particularly prominent in individuals who excel at letter‐based N‐back tasks (Zou et al. 2013), suggesting that the FC between these regions may play a role in supporting WM processes, even in the absence of explicit task demands. Extending this understanding, our findings reveal an additional positive correlation between verbal d′ and connectivity between right IPL and left SPL. These areas are well‐established as critical for shifting attention to behaviorally relevant stimuli, a process essential for effective WM (Berryhill et al. 2011; d'Esposito et al. 1998). The SPL, in particular, has been highlighted in lesion studies for its role in the manipulation of information, further underscoring its importance in complex cognitive operations (Koenigs et al. 2009). The IPL is implicated in the storage component of the phonological loop and more complex executive operations (Marshuetz et al. 2000). It is thought to be vital for encoding and storing temporal order information (Marshuetz et al. 2000), reactivating sources of information in neural regions through attentional mechanisms, and facilitating rapid shifts of attention (LaBar et al. 1999).

Spatial WM has been consistently linked to regions such as the dlPFC, the MFG, and the superior frontal cortex (SFC) (Courtney et al. 1998; Glahn et al. 2002; Ren et al. 2019; van Dam et al. 2015). Furthermore, the lateral PFC is well recognized for its role in spatial WM processing (Owen et al. 1996). Specifically, the MFG and ITG are acknowledged for their roles in visual WM, particularly in pattern recognition and the encoding of behaviorally relevant stimuli, processes integral to the spatial N‐back task employed in our study (Wager and Smith 2003). Interestingly, although spatial WM is frequently associated with right‐lateralized visuospatial processing, our study observed rs‐connectivity also in left‐hemisphere (LH) regions such as the PMC, SMA, and SPL during nonverbal WM tasks. These findings align with prior studies showing involvement of LH in spatial WM (Champod and Petrides 2007; Paulraj et al. 2018; Pollmann and Yves Von Cramon 2000). Moreover, transcranial magnetic stimulation studies, have demonstrated that disrupted motor activity in the left PMC impairs performance in nonverbal rehearsal, further supporting a connection between WM and motor‐related regions of FPN (Liao et al. 2014). Consistent with our findings of cross‐hemispheric rs‐functional interactions, the premotor and posterior parietal cortices of both hemispheres appear to be positively associated with spatial WM.

Despite evidence provided by task‐based fMRI studies about the involvement of PMC, preSMA, and SMA in WM (Wager and Smith 2003), their rs‐FC patterns are less investigated. Our study addresses this gap by bringing forward that rs‐FC between these motor planning‐related regions are associated, both positively and negatively, to verbal and spatial WM. This finding aligns with previous solid results showing that WM maintenance and manipulation are supported not only by the dorsal and ventral PFC but also by the dorsal premotor regions (LaBar et al. 1999; Rottschy et al. 2012).

### 4.2 A Sensorimotor Approach to WM

Based on evidence provided by neuroimaging, experimental and developmental studies we argued that the FPN evolved from regions originally specialized for motor planning to more general networks supporting higher cognitive functions such as attention and WM (Ptak et al. 2017). Activation fMRI studies show that frontoparietal regions associated with WM maintenance appear to play a determinant role in motor planning and preparation, rather than the execution of movement (Hülsmann et al. 2003). Certain speculations can be put forward for contextualizing the relationship between motor planning and WM. One hypothesis is that motor‐related regions contribute to the maintenance of verbal and nonverbal information in WM by generating motor traces that support active rehearsal processes (Marvel et al. 2019; Marvel and Desmond 2012). According to this view, the PFC does not hold active representations of WM items in a mental repository but selects appropriate memory‐guided responses for goal‐directed actions (Pochon et al. 2001). Thus, these motor‐related areas could be part of a network focused on action preparation using WM information, rather than on the rehearsal and storage of sensory details (Nobre and Stokes 2019; Olivers and Roelfsema 2020).

We propose that the contribution of motor‐related regions to WM is closely linked to their role in the internal simulation and replay of motor actions, a function that could have been reused to support covert cognitive operations (Ptak et al. 2021). This proposal is similar to the dorsal‐executive hypothesis, which posits that motor‐related regions within dorsal FPN, including the SFC and dlPFC, are essential for continuous updating and temporal order memory (Wager and Smith 2003). Findings that connectivity between these regions predicts performance in complex WM tasks, such as sequence reordering and monitoring multiple items, support this hypothesis (Champod and Petrides 2007; Chein and Fiez 2001). A particularly strong link appears to exist between motor simulation and the updating of contents in WM, as both require dynamic monitoring and refreshing of information, a process crucial for the sequencing of individual movements and ordering items in WM.

## 5 Conclusions

Our research suggests that brain regions involved in motor planning are also associated with WM and may play a broader role in underlying higher‐order cognitive processes, such as updating. These findings challenge the view that these regions are solely involved in motor preparation, indicating that cognitive mechanisms required for motor planning (e.g., internal replay and simulation of motor processes) may also contribute to high‐level cognitive tasks. We are aware, that while the observed correlations indicate association, they do not establish causality. Instead, the connectivity patterns identified in our study suggest the likelihood of involvement of these regions in supporting the behavior of interest. Combining task‐based or causal methodologies (e.g., TMS or lesion studies) with the rs‐FC paradigm would help confirm the specific roles of these regions. Overall, this research contributes to the growing body of evidence that cognitive processes are deeply rooted in the brain's motor and sensory functions.

## Author Contributions

**Emilie Marti**: data curation, formal analysis, investigation, methodology, project administration, visualization, writing–original draft, writing–review and editing. **Sélim Coll**: data curation, formal analysis, investigation, methodology, project administration, supervision, validation. **Naz Doganci**: data curation, formal analysis, investigation, methodology, project administration. **Radek Ptak**: conceptualization, data curation, formal analysis, funding acquisition, investigation, methodology, project administration, resources, supervision, validation, writing–review and editing.

### Peer Review

The peer review history for this article is available at https://publons.com/publon/10.1002/brb3.70376.

## Data Availability

The data that support the findings of this study are available from the corresponding author upon reasonable request.
